# Supplementary material for: Development and In Vitro–In Vivo Correlation Evaluation of IMM-H014 Extended-Release Tablets for the Treatment of Fatty Liver Disease
Source: Int J Mol Sci. 2023 Aug 2;24(15):12328. doi: 10.3390/ijms241512328 (PMC10418331; doi:10.3390/ijms241512328)
Supplement: Supplementary file 1 [file ijms-24-12328-s001.zip › ijms-2516882-supplementary.pdf]

Supporting information

Development and In Vitro–In Vivo Correlation Evaluation of IMM-H014 Extended-Release Tablets for the Treatment of Fatty Liver Disease

Table of contents

Table S1. Saturated solubility of IMM-H014 in different media 2

Table S2. Cumulative percentage drug release of IMM-H014 tablet with different matrix materials (F1 to F4)..3

Figure S1. In vitro release profiles of tablet with different matrix materials (F1 to F4) in 0.01N HCl →pH 7.5 PBS medium 4

Table S3. Cumulative percentage drug release of IMM-H014 tablet with HPMC concentration (IR, F5 to F10) (n=6)……………………………………………………………5

Table S4. Pharmacokinetic parameters of ER with different HPMC concentration and IR.6

Table S5. Cumulative percentage drug release of IMM-H014 ER tablets in five different dissolution media...7

Figure S2. The in vitro release behaviors: IMM-H014 ER tablets in five different dissolution media; 0.01 N HCl→pH7.5 phosphate buffer saline （A）, 0.01 N HCl（B）, pH 4.5 acetate buffer extraction procedure （C）, water（D）, pH 6.8 phosphate buffer saline（E）; three batches of IMM-H014 ER tablets (n = 12)...10

Table S1. Saturated solubility of IMM-H014 in different media (n=3)

pH	Saturation solubility (mg/mL) ±SD
2.0	229.1±8.7
4.5	32.5±1.1
6.8	5.0±0.2
12.0	0.0

Table S2. Cumulative percentage drug release of IMM-H014 tablet with different matrix materials (F1 to F4) (n=6)

Time (h)	IMM-H014±SD（%）			
	**F1**	**F2**	**F3**	**F4**
0.5	3.4±0.8	1.4±0.3	6.1±0.4	11.9±8.7
1	7.3±1.5	2.3±0.5	12.0±0.5	19.7±10.4
2	14.8±2.3	4.5±0.9	22.2±1.1	29.0±11.4
3	22.1±2.9	7.1±1.4	30.0±1.8	36.1±11.9
4	29.1±3.4	9.7±1.9	36.8±2.1	41.6±11.6
6	41.8±3.9	14.3±2.6	49.0±2.7	50.2±11.5
8	54.5±4.2	18.8±3.1	60.7±3.0	57.8±11.1
10	65.4±3.7	22.7±3.4	69.7±2.4	64.1±10.0
12	74.8±2.9	26.3±3.4	76.9±2.3	69.4±8.3
16	86.7±2.3	32.5±3.4	85.8±2.5	78.0±5.0
20	93.9±1.8	37.6±3.1	89.1±3.1	84.8±3.3
24	96.4±1.2	42.1±2.8	90.1±2.5	89.6±4.2

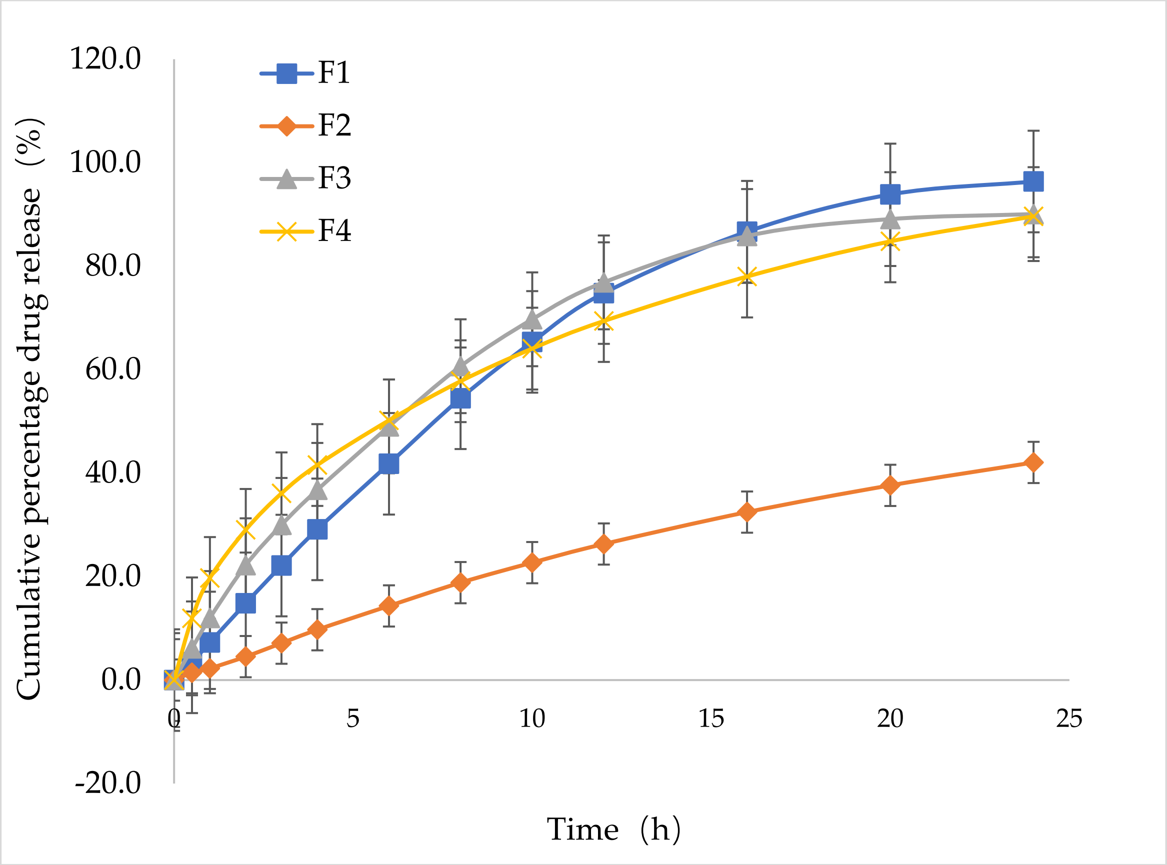

Figure S1. In vitro release profiles of tablet with different matrix materials (F1 to F4) in 0.01N HCl →pH 7.5 PBS medium (n=6)

Table S3. Cumulative percentage drug release of IMM-H014 tablet with HPMC concentration (IR, F5 to F10) (n=6)

Time (h)	IMM-H014±SD（%）						
	IR	F5	F6	F7	F8	F9	F10
0	0.0	0.0	0.0	0.0	0.0	0.0	0.0
0.08	62.3±3.3	/	/	/	/	/	/
0.17	91.0±2.4	/	/	/	/	/	/
0.25	95.0±3.1	/	/	/	/	/	/
0.33	96.4±2.2	/	/	/	/	/	/
0.5	97.6±2.1	10.2±0.9	12.6±0.9	13.2±1.1	13.9±0.6	32.8±1.2	46.4±3.5
0.75	98.5±1.8	/	/	/	/	/	/
1	99.5±1.5	18.6±0.6	20.7±1.7	23.8±1.8	23.9±0.7	49.6±2.9	71.9±3.3
1.5	99.6±1.3	/	/	/	/	/	/
2	99.7±1.2	29.1±0.0	33.1±2.3	39.2±2.8	39.0±0.8	72.5±3.7	99.6±2.4
3	99.7±0.6	32.4±0.5	34.7±2.3	40.7±3.0	43.2±1.2	73.3±2.7	95.1±3.5
4	99.8±0.6	33.4±0.5	37.7±2.4	44.3±3.3	48.5±1.3	79.6±2.7	97.1±3.7
6	99.8±0.7	37.1±1.1	44.0±2.4	51.6±4.4	57.5±2.5	88.2±3.0	97.6±3.7
8	/	41.4±2.1	51.3±2.3	59.7±5.2	64.0±7.0	91.3±4.3	97.8±3.8
10	/	46.1±3.2	58.9±2.4	67.5±5.7	72.6±7.6	94.3±3.8	98.3±3.8
12	/	51.1±4.4	66.0±2.6	74.5±6.0	80.3±6.0	95.5±4.2	98.4±3.7
16	/	61.0±6.2	77.5±3.4	84.4±5.6	86.2±6.6	96.8±4.7	99.1±3.8
20	/	69.8±6.6	85.7±3.6	91.0±4.6	93.0±4.7	96.9±4.6	99.2±3.9
24	/	77.2±6.4	91.1±3.3	95.4±3.8	96.9±1.5	97.1±4.8	99.4±3.9

Table S4. Pharmacokinetic parameters of ER with different HPMC concentration and IR (n=6)

**Pharmacokinetic parameters**	**IR**	**F5**	**F6**	**F7**	**F8**	**F9**	**F10**
AUC(0-t) (ng·h/m) ±SD	11415.9± 1986.0	6691.7± 1969.1	7377.1± 3260.9	8395.1± 2432.0	9894.3± 3447.4	10980.8± 1663.7	10209.2± 2290.2
AUC(0-∞) (ng·h/m) ±SD	11545.2± 2008.7	7034.5± 2072.1	7904.8± 3506.0	9480.3± 2507.7	10508.1± 3692.7	11393.3± 1729.1	10579.6± 2358.2
MRT(0-t) (h)±SD	4.2±0.6	7.2±1.3	7.7±0.5	8.1±0.9	6.8±1.7	7.2±1.2	6.4±0.9
MRT(0-∞) (h) ±SD	4.5±0.9	8.4±1.4	9.5±1.6	10.9±1.2	8.1±2.0	8.0±1.2	7.4±1.3
Tmax (h)±SD	0.5±0.0	3.1±2.4	2.3±1.9	2.75±1.5	1.6±1.1	3.1±2.9	2.2±0.8
Cmax (ng/mL) ±SD	4447.8± 916.5	739.4± 173.4	798.1± 708.7	781.6± 232.0	1326.5± 304.2	1276.8± 322.6	1553.9± 418.8
F（%）	100%	58.6	64.6	73.5	86.7	96.2	89.4

Table S5. Cumulative percentage drug release of IMM-H014 ER tablets in five different dissolution media (n=12)

Time (h)	IMM-H014 (%)±SD														
	0.01M HCl→pH7.5 PBS			0.01M HCl			pH4.5 PBS			water			pH6.8 PBS		
	1	2	3	1	2	3	1	2	3	1	2	3	1	2	3
0	0	0	0	0.0	0.0	0.0	0.0	0.0	0	0.0	0.0	0	0.0	0.0	0
0.5	20.2±0.9	19.8±1.6	20.4±1.3	20.0±1.2	19.7±1.4	19.9±1.4	17.9±1.6	18.1±1.7	17.6±1.7	19.9±1.8	18.7±1.4	19.5±1.8	10.0±2.3	8.8±2.2	7.6±1.5
1	33.7±1.1	33.2±2.0	33.5±2.0	33.0±1.7	32.3±1.6	32.8±2.0	29.5±2.2	29.6±2.4	29.7±1.9	32.5±2.4	31.2±1.7	30.1±1.6	17.9±3.5	16.5±3.3	14.5±2.6
2	52.2±1.6	51.5±2.4	51.1±2.8	51.0±2.3	49.7±1.9	49.6±2.7	45.5±3.1	45.6±3.3	45.8±2.4	49.4±3.3	47.4±2.5	45.0±2.0	32.2±4.6	29.7±4.8	25.8±3.8
3	54.4±2.1	53.8±2.6	52.2±2.8	64.8±2.7	62.9±2.2	61.9±3.1	58.8±4.0	58.6±4.2	58.8±2.9	62.8±4.4	60.3±3.1	56.9±2.5	44.0±5.2	41.0±5.8	35.7±4.3
4	62.4±2.7	62.4±3.1	58.7±3.3	75.9±3.2	73.7±2.5	71.8±3.5	70.1±4.9	69.6±5.1	69.5±3.3	73.7±5.2	70.9±3.9	66.1±3.1	53.9±5.7	50.8±6.5	44.3±4.7
6	75.3±3.1	75.9±3.6	69.7±4.3	91.6±3.4	89.3±2.6	86.6±3.9	87.4±5.9	87.8±6.2	86.6±4.1	88.8±5.8	86.1±4.6	82.7±3.7	70.4±5.9	66.9±7.1	60.1±5.9
8	84.9±2.4	85.7±3.3	79.5±5.1	97.9±2.8	96.5±1.6	95.0±3.3	96.3±2.5	96.1±3.6	97.1±4.3	95.3±3.8	93.9±3.8	91.0±3.3	83.3±5.6	80.2±7.2	73.3±6.6
10	90.7±1.2	90.9±2.2	86.8±4.4	99.1±2.3	97.7±1.3	97.4±2.8	98.5±1.0	98.3±1.4	100.6±3.2	97.5±2.5	96.3±2.8	97.1±2.1	91.1±4.5	88.4±5.7	84.1±6.2
12	93.7±1.1	92.3±2.1	90.9±3.4	99.2±2.1	97.8±1.2	97.8±2.8	98.8±1.2	98.8±1.2	100.8±3.3	97.6±1.9	97.1±2.4	99.3±1.4	95.0±3.0	93.2±3.9	89.6±5.0
16	94.8±1.4	93.3±2.3	94.4±2.5	99.3±2.4	98.0±1.3	98.1±2.9	98.8±1.3	98.7±1.1	99.9±3.6	98.8±1.8	97.7±2.1	100.2±1.6	99.4±1.3	97.5±2.3	92.4±3.1
20	95.2±1.5	93.2±2.4	95.0±2.4	99.4±2.5	98.0±1.3	98.2±3.1	98.5±1.3	98.3±1.1	100.8±2.2	97.8±1.7	97.8±2.3	99.3±1.8	98.8±0.8	98.3±2.1	94.8±2.0
24	95.5±1.6	93.3±2.5	95.0±2.5	99.7±2.5	98.1±1.4	98.4±3.1	98.5±1.3	98.4±1.1	100.9±2.1	98.4±1.7	98.0±2.3	100.0±1.8	98.9±0.8	98.2±2.3	97.7±1.6

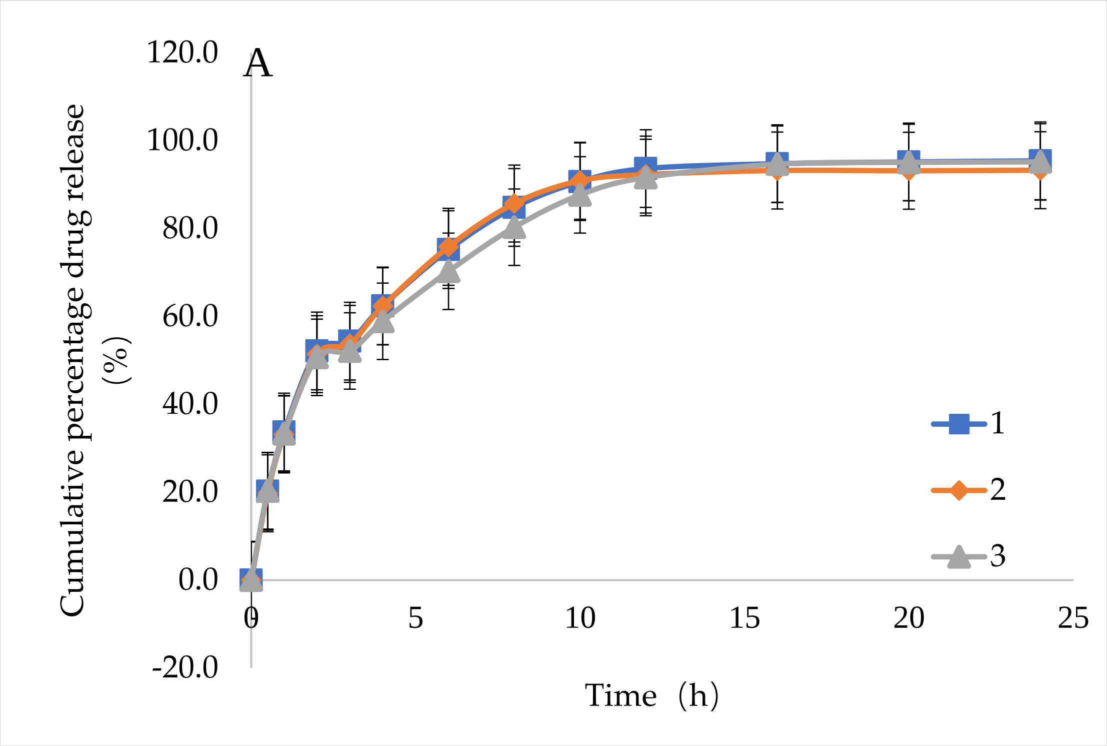
A
Cumulative percentage drug release
(%)
120.0
100.0
80.0
60.0
40.0
20.0
0.0
-20.0
0
5
10
15
20
25
Time (h)
1
2
3

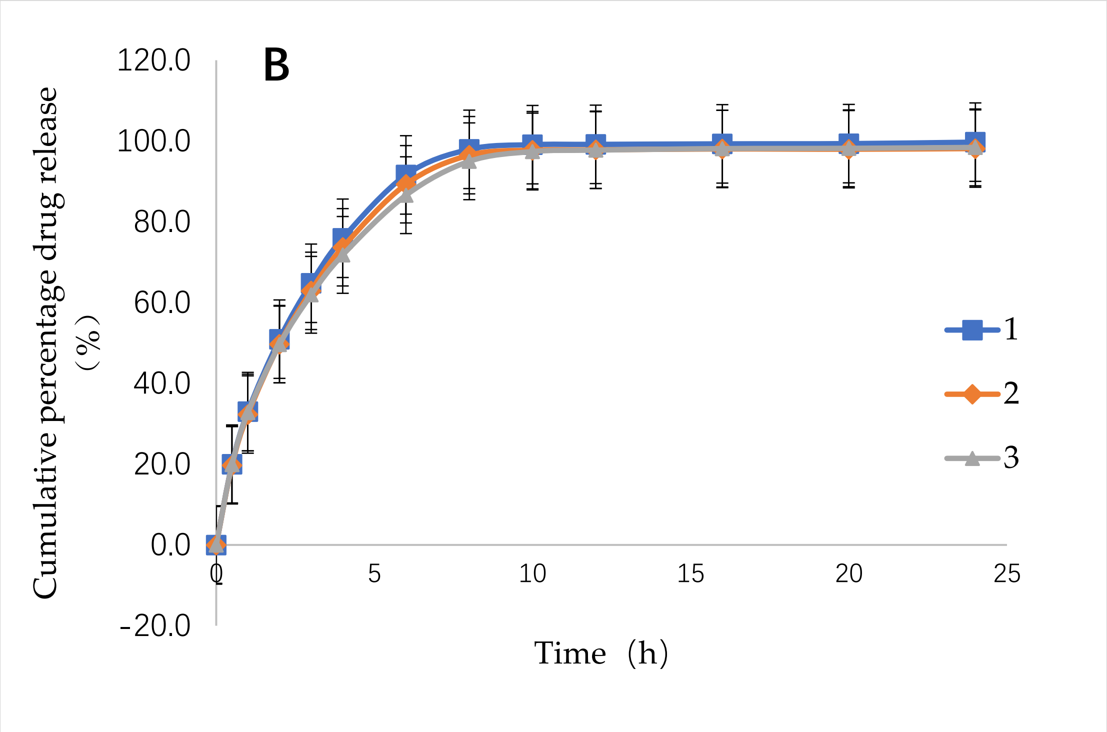
B
Cumulative percentage drug release
(%)
120.0
100.0
80.0
60.0
40.0
20.0
0.0
-20.0
0
5
10
15
20
25
Time (h)
1
2
3

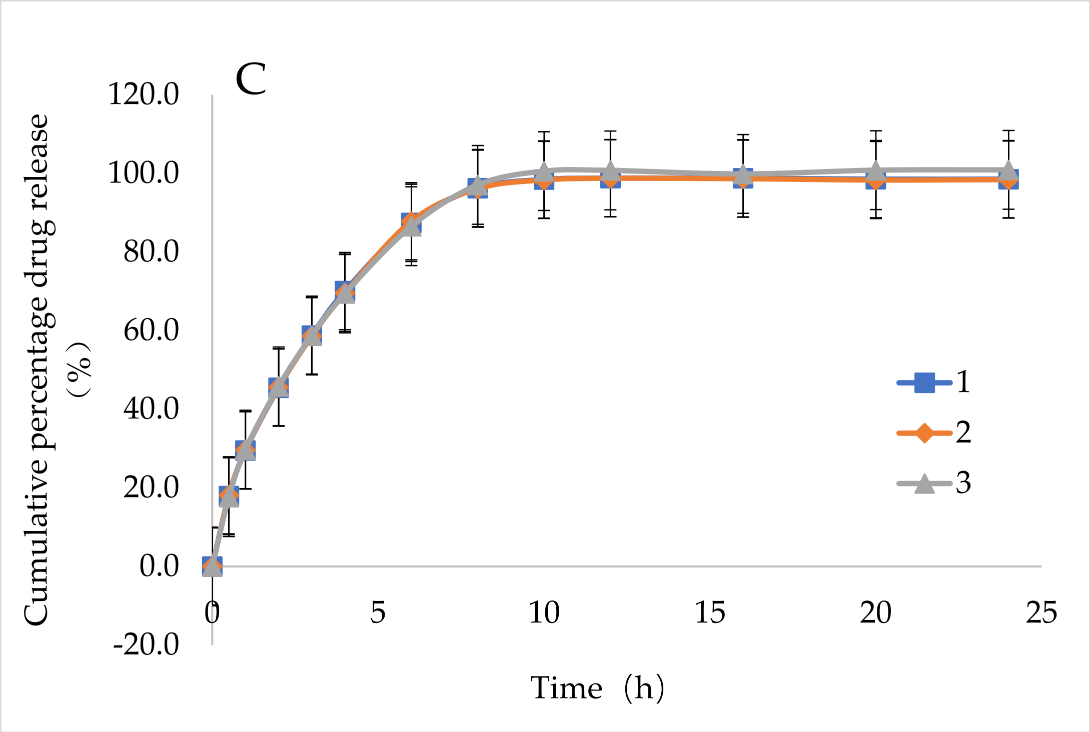
C
Cumulative percentage drug release （%）
120.0
100.0
80.0
60.0
40.0
20.0
0.0
-20.0
0
5
10
15
20
25
Time （h）
1
2
3

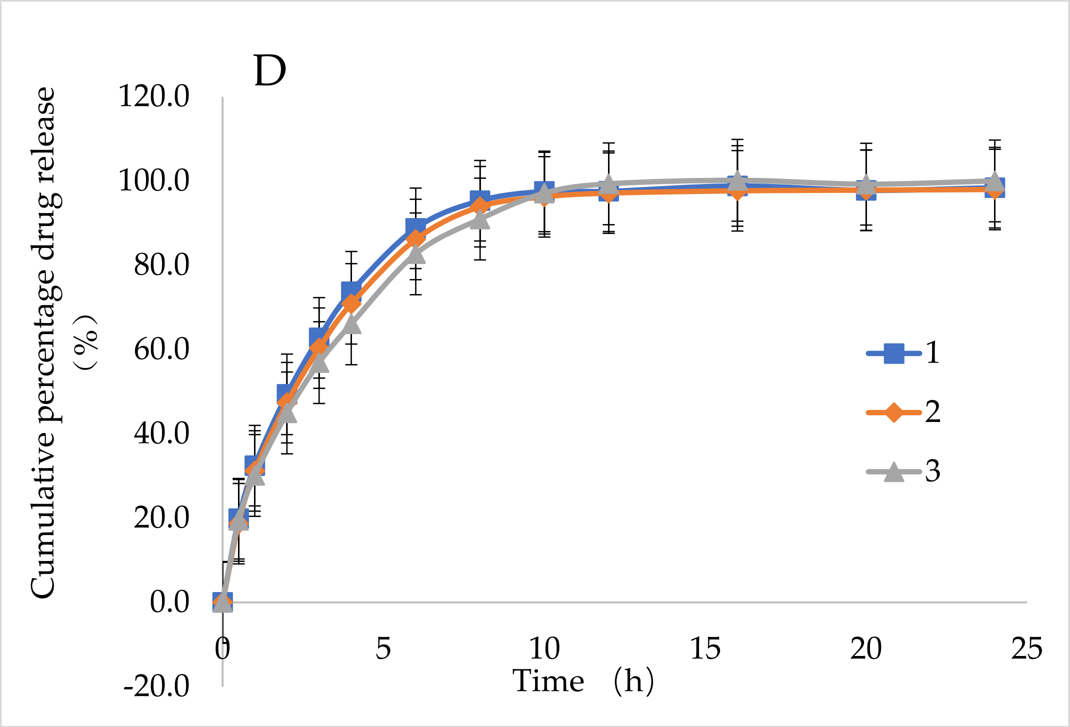
D
Cumulative percentage drug release （%）
120.0
100.0
80.0
60.0
40.0
20.0
0.0
-20.0
0
5
10
15
20
25
Time （h）
1
2
3

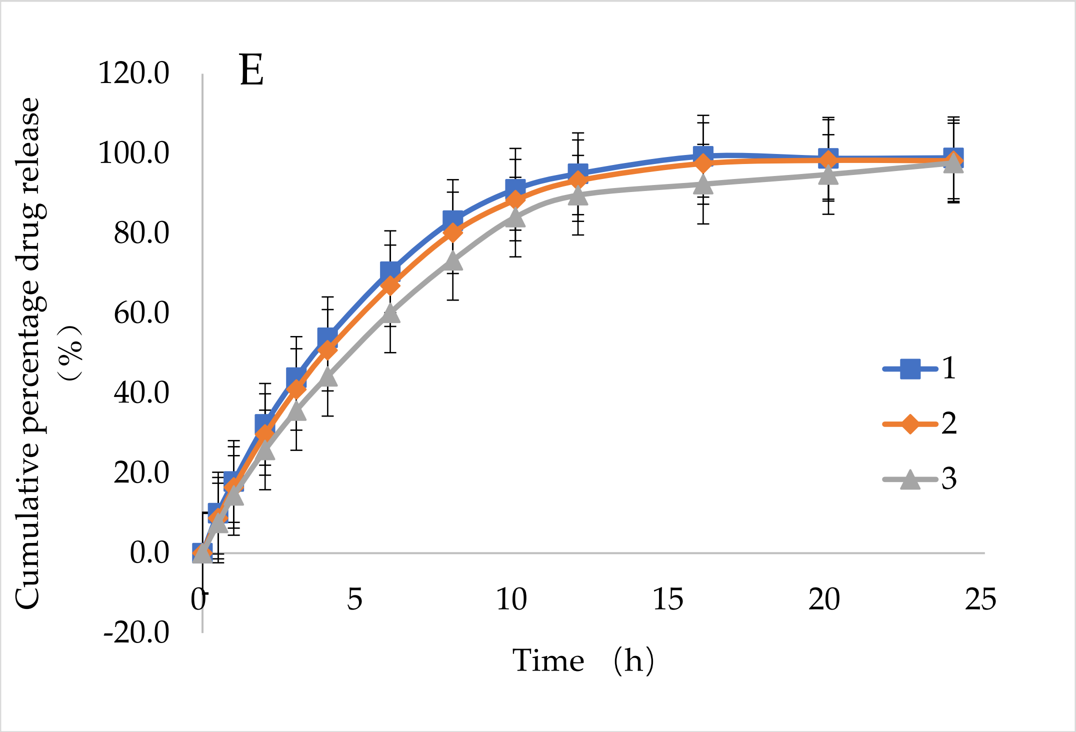

Figure S2. The in vitro release behaviors: IMM-H014 ER tablets in five different dissolution media; 0.01 N HCl→pH7.5 phosphate buffer saline （A）, 0.01 N HCl（B）, pH 4.5 acetate buffer extraction procedure （C）， water（D）， pH 6.8 phosphate buffer saline（E）; three batches of IMM-H014 ER tablets (n = 12)
